# Biochemical Characterization of Traditional Varieties of Sweet Pepper (*Capsicum annuum* L.) of the Campania Region, Southern Italy

**DOI:** 10.3390/antiox9060556

**Published:** 2020-06-26

**Authors:** Florinda Fratianni, Antonio d’Acierno, Autilia Cozzolino, Patrizia Spigno, Riccardo Riccardi, Francesco Raimo, Catello Pane, Massimo Zaccardelli, Valentina Tranchida Lombardo, Marina Tucci, Stefania Grillo, Raffaele Coppola, Filomena Nazzaro

**Affiliations:** 1 Institute of Food Science, CNR-ISA, Via Roma 64, 83100 Avellino, Italy; filomena.nazzaro@isa.cnr.it; 2 Department of Agricultural, Environmental and Food Sciences (DiAAA)-University of Molise, Via de Sanctis snc, 86100 Campobasso, Italy; autilia.cozzolino@unimol.it (A.C.); coppola@unimol.it (R.C.); 3 Cooperativa “ARCA 2010”, Via Varignano 7, 8100 Acerra (NA), Italy; patspigno@hotmail.com (P.S.); ricc.riccardi@libero.it (R.R.); 4 Horticulture Research Center (CRA-ORT), Via Cavalleggeri 25, 84098 Pontecagnano Faiano (SA), Italy; francesco.raimo@crea.gov.it (F.R.); catello.pane@crea.gov.it (C.P.); massimo.zaccardelli@crea.gov.it (M.Z.); 5 Institute of Biosciences and Bioresources, CNR-IBBR, O.U. of Portici (NA), Via Università 100, 80055 Portici (NA), Italy; valentinatranchida@tiscali.it (V.T.L.); mtucci@unina.it (M.T.); grillo@unina.it (S.G.)

**Keywords:** biodiversity, *Capsicum annuum* L., antioxidant activity, β-carotene, ascorbic acid, polyphenols, statistical analysis

## Abstract

Bioactive compounds of different Campania native sweet pepper varieties were evaluated. Polyphenols ranged between 1.37 mmol g^−1^ and 3.42 mmol g^−1^, β-carotene was abundant in the red variety “Cazzone” (7.05 μg g^−1^). Yellow and red varieties showed a content of ascorbic acid not inferior to 0.82 mg g^−1^, while in some green varieties the presence of ascorbic acid was almost inconsistent. Interrelationships between the parameters analyzed and the varieties showed that ascorbic acid could represent the factor mostly influencing the antioxidant activity. Polyphenol profile was different among the varieties, with a general prevalence of acidic phenols in yellow varieties and of flavonoids in red varieties. Principal Component Analysis, applied to ascorbic acid, total polyphenols and β-carotene, revealed that two of the green varieties (“Friariello napoletano” and “Friariello Sigaretta”) were well clustered and that the yellow variety “Corno di capra” showed similarity with the green varieties, in particular with “Friariello Nocerese”. This was confirmed by the interrelationships applied to polyphenol composition, which let us to light on a clustering of several red and yellow varieties, and that mainly the yellow ”Corno di capra” was closer to the green varieties of “Friariello”.

## 1. Introduction

Sweet pepper (*Capsicum annuum* L.), belonging to the Solanaceae family, is one of five cultivated species of the genus *Capsicum*, including sweet and hot peppers. It is a component present in the diet of many populations in the world; as such, therefore it represents an important source of income for farmers and operators in the agro-industrial sector. *C. annuum* L., originated from Central and South America and arrived in Europe in the sixteenth century with the Spanish and Portuguese expeditions to the lands of the New World. Once introduced in cultivation, pepper soon became a very common vegetable in the kitchens of all the countries of Europe. Today, *C. annuum* L. is cultivated in a large number of varieties all over the world, reaching a cultivated area that exceeds 1.5 million hectares. Italy is one of the most important countries in the world for this crop. The commercial produce of sweet pepper is the fruit, having different forms and size, as well as several colors, which range from yellow to red, from intense purple to dark green to black, depending on cultivar, maturity, growing conditions, and postharvest manipulation. Sweet pepper represents an excellent source of several antioxidant molecules, in particular carotenoids [1,2] ascorbic acid, and polyphenols, such as quercetin [3], which received huge interest for to their antioxidant properties [4]. A considerable weight of evidence suggests that consumption of fruit and vegetables is beneficial for human health and may help in preventing several chronic diseases [5]. Since last decades, secondary metabolites of vegetables have come into light for their presumed role in fighting cancer and cardiovascular diseases [6,7] as well as in slowing down aging and atherosclerosis [8].

Campania is among the most prominent regions of Europe in terms of production and number of varieties of fruit and vegetables [9]. Over the millennia, indigenous plant varieties, in a sort of ideal symbiosis with the territory, have managed to express their quality and nutritional value at the best. Several of these varieties are still widespread, while others are cultivated as niche products, or have been abandoned, supplanted by commercial varieties, which are more productive but often have lower quality. Pepper is one of the most important crops for this area and several varieties have become traditional of the Campania region, giving very worthy inputs to its rich cultivated biodiversity. Plant biodiversity includes the enormous amount of vegetal germplasm differentiated in the course of the long history of biological evolution of species. In agriculture, biodiversity is also the work of human selection from a “wild” gene pool to obtain breeds and varieties adapted to various ecological, economic and social conditions. Phytochemicals of sweet peppers undoubtedly arouse great interest mainly act as antioxidant agents. They represent also important element for human health and can prevent the occurrence of disease linked to oxidative stress, including cardiovascular and neurodegenerative diseases, and cancer [10]. Certain green, red, orange, and yellow pepper showed interesting capacity to inhibit some key enzymes linked to Alzheimer disease [acetylcholinesterase (AChE), butyrylcholinesterase (BChE), and β-secretase (BACE1)] [11]. Due to the broad biochemical and nutritional variations existing among the different varieties within each plant species, the identification of the best genotypes assumes a particular importance for both breeders and consumers, which thereby can select and consume products with high nutritional quality, respectively. Keeping this in view, the present study focused on the following objectives: (a) to analyze the contents of bioactive compounds of native pepper varieties from the Campania region, Southern Italy, including β-carotene, total phenolics, phenolic profiles, ascorbic acid; (b) to determine their in vitro antioxidant activities through the DPPH radical-scavenging activity; (c) to correlate the antioxidant activity of the varieties to total phenolics, ascorbic acid and carotenoids, with the aim of identifying the main factors influencing the antioxidant properties of the product. Interrelationships between the parameters analyzed and the different varieties were investigated by principal component analysis (PCA).

## 2. Materials and Methods

### 2.1. Chemicals

Caffeic, ferulic, p-coumaric, gallic, chlorogenic acids, epicatechin, rutin, quercetin, 2,2-diphenyl-1-picrylhydrazyl (DPPH), β-carotene, ascorbic acid, HPLC-grade methanol, sulphuric, metaphosphoric, acetic and formic acids, acetonitrile, petroleum ether, ethanol and acetone were purchased from Sigma-Aldrich (Milano, Italy). Apigenin was purchased from Extrasynthese (Genay, France). The Folin–Ciocalteu reagent was purchased from BIO-RAD (Milano, Italy). Water was distilled and filtered through a Milli-Q apparatus (Millipore, Milano, Italy) before use.

### 2.2. Plant Material

**Figure 1:**
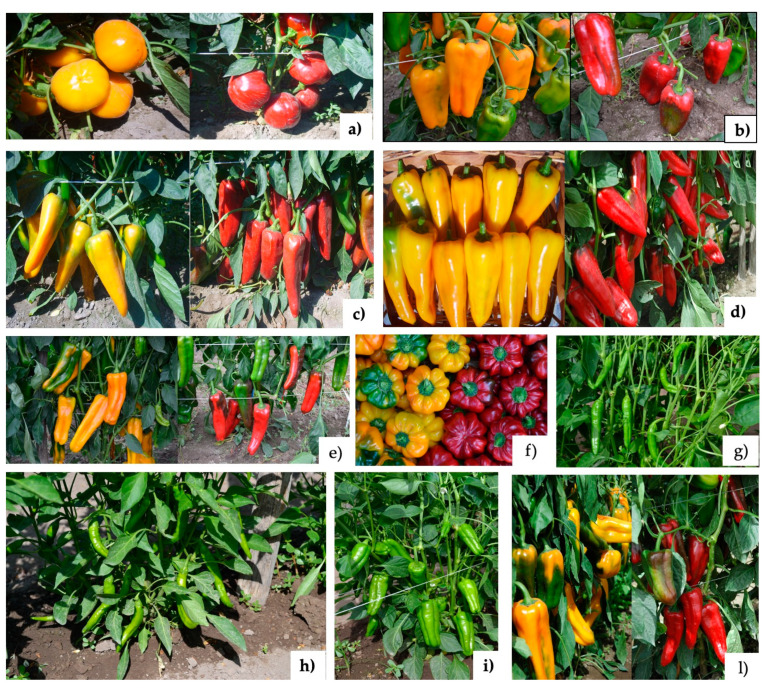
Traditional sweet pepper varieties of the Campania region analyzed in the present work. Legend: (**a**) Papaccella liscia; (**b**) Sassaniello; (**c**) Cornetto di Acerra; (**d**) Corno di capra; (**e**) Corno Marconi (**f**) Papaccella Napoletana; (**g**) Friariello a sigaretta; (**h**) Friariello Napoletano; (**i**) Friariello Nocerese; (**l**) Cazzone. The photos were made by the authors.

Plant material (Figure 1) included seven types of yellow and red sweet pepper, (*Capsicum annuum* L.) (“Papaccella napoletana”, “Papaccella liscia”, “Corno Marconi”, “Corno di capra”, “Cornetto di Acerra”, “Cazzone”, “Sassaniello”), corresponding to fourteen varieties, and three varieties of green sweet pepper (“Friariello napoletano”, “Friariello nocerese”, “Friariello a sigaretta”) listed by the Official Bulletin of the Campania Region (B.U.R.C. n°42, 145, 2009), grown and collected in the farm of the “Cooperativa ARCA 2010” sited in Acerra (NA), Italy. Acerra (40.9441° N, 14.3714° E) is characterized by a Mediterranean climate with an average of air temperature (*T*), humidity (*U*) and rainy days (*R*) *T* = 22.7 °C; *U* = 63.8%; *R* = 6.6 during the growing season [12]. The seeds of the varieties of pepper are stored and preserved by the gene bank “banca del germoplasma” at the “Consorzio Arca 2010” on behalf of the Campania region. Seedlings were transplanted in three rows with 60 plants (“Cazzone” and “Sassaniello”); 100 plants (“Friariello”); 83 plants (“Papaccella napoletana”, “Papaccella napoletana liscia”, “Corno di capra”). Cultivation techniques included stakes (1.2 m) as support and twine threads to tie the plants. Microirrigation was used as technique of irrigation. Replicates were 3. Each replicate included 10 plants collected for the determination of marketable production. Four harvests were generally performed from the beginning of August to the beginning of October. Before analysis, fruits were gently cleaned. Peduncles and seeds of pepper fruits were removed; the comestible portion was cut and immediately stored at −26 °C.

### 2.3. Dosage of Ascorbic Acid

Dosage of ascorbic acid was performed following the method of Nazzaro et al. [13]. All samples were cut, squeezed and incubated in three volumes of metaphosphoric acid (4%) and maintained for 1 h at 4 °C, avoiding the exposition to the light. Extracts were subjected to centrifugation (11,600× *g* for 10 min at 4 °C, Biofuge, Beckman Italia, Cassina de’ Pecchi, Milano, Italy), and filtration (0.45 μm mesh, Millipore, Milano, Italy) to recovery the supernatant. A Gold System chromatograph equipped with an UV detector (Beckman Italia, Cascina dè Pecchi MI, Italy) and a Khromasil KR 100-5 C_18_ column (25 cm × 4.6 mm) was used to assess, through RP-HPLC, the amount of ascorbic acid present in the samples (run condition = mobile phase: sulphuric acid 0.001 M in HPLC-grade water; injection volume: 20 μL; flow rate: 1.0 mL min^−1^; detection wavelength: 245 nm; Temperature: room temperature). Ascorbic acid, previously dissolved in the mobile phase, was used as standard to generate the standard curve.

### 2.4. Carotene Content

Extraction was carried out according to the method described by Nazzaro et al. [13], modified as follows: fresh sample was cut and squeezed in ethanol (1:1 *w/v*), and then petroleum ether was added (1.5:1 *v/v*). The mixture was vigorously shaken; the supernatant was recovered by centrifugation (11,600× *g*, 15 min; Biofuge, Beckman Italia, Milano, Italy). The steps were repeated until the complete disappearance of the color, then the supernatants were put together. The amount of carotenoids was evaluated at 450 nm with petroleum ether as blank, and using the extinction coefficient ε = 2592, using the spectrophotometer Cary 50 Uv/Vis (Varian-Agilent Italia, Cernusco sul Naviglio, Italy).

### 2.5. Total Polyphenols and Antioxidant Activity

Samples were cut and squeezed (1:3 *w/v*) in methanol (containing acetic acid 1%) overnight at 4 °C. After centrifugation (11,600× *g*, 15 min; Biofuge, Beckman Italia), supernatants were recovered and the polyphenols amount and profile as well as the antioxidant activity was evaluated. The content of total polyphenols was spectrophotometrically evaluated (Cary 50 Varian-Agilent Italia) at λ = 760 nm, following the method described by Singleton and Rossi [14] with Folin Ciocalteau reagent. Gallic acid was used as standard. Results were indicated as mMol gallic acid equivalent g^−1^ of fresh sample. Radical-scavenging activity was assayed through the use of the stable radical 2,2-diphenyl-1-picrylhydrazyl (DPPH assay) following the method of Ombra et al. [15]. The analysis was performed in microplates by adding 15 μL of extract to 300 μL of a methanol- DPPH solution (6 × 10^–5^ M). The absorbance was measured at λ = 517 nm (Cary 50 MPR Varian-Agilent Italia). The scavenging activity was expressed in terms of EC_50_, indicating the amount of sample amount (mg) necessary to inhibit DPPH radical activity by 50% for the duration of 60 min of incubation.

The experiments were performed in triplicate. Results were expressed as the mean values ± standard deviation.

### 2.6. Chromatographic Analysis

Ultra-high-performance liquid chromatography (UPLC) analysis was made using the ACQUITY Ultra Performance LC^TM^ system (Waters, Milford, MA, USA) connected to a PDA 2996 photodiode array detector (Waters), characterized by a low dispersion with enlargement of the band lower than 10 μL; automatic control of the temperature; technology Smart-Start for the gradient, to perform a controlled mixing of the solvents until pressure = 15,000 psi; controlled degassing and automatized firmness of the solvents; direct setting of gradients in terms of pH, molarity and/or organic composition. Detector PDA: linear answer in the interval of wavelength ranging between 190 nm and 500 nm and for values of absorbance until 2.0 AU: Deviation of 1.3% at 2.0 AU; Deviation at 5.0% at 2.8 AU. The acquisition and processing of the relative data, as well as the control of the instruments was performed through the Empower software. The analysis was performed following the method described by Fratianni et al. [16] and Pane et al. [17]. All the extracts and standards were dissolved in methanol, and filtered through Whatman 0.45 μm (Waters, Milford, MA, USA). The analyses were performed at 30 °C. Running conditions = Injection volume: 5 μL. Mobile phase: solvent A (7.5 mMol acetic acid) and solvent B (acetonitrile); flow rate: 250 μL min^−1^; column: reversed phase column (BEH C_18_, 1.7 μm, 2.1 × 100 mm Waters, based on ethyl bridge silanes, stable in a range of pH between 2 and 12 and temperatures up to 90 °C). The analysis was performed with a gradient elution (5% B for 0.8 min; 5–20% B over 5.2 min; 20% B for 0.5 min; 20–30% B for 1 min; 30% B for 0.2 min, 30–50% B over 2.3 min, 50–100% B over 1 min, 100% B for 1 min; 100–5% B over 0.5 min). Finally, the column was restored to the initial conditions for 2.5 min. Conditions of the apparatus= Pressure ranging from 6000–8000 psi; Scanning range of LC detector: 210–400 nm, resolution: 1.2 nm).

### 2.7. Statistical Analysis

Data were expressed as mean ± standard deviation of triplicate measurements. The PC software “Excel Statistics” was used for the calculations. Interrelationships between the parameters analyzed and the different varieties were investigated by principal component analysis (PCA), following the method of Fratianni et al. [18] and using the software package MATLAB.

## 3. Results and Discussion

### 3.1. Ascorbic Acid Content

Ascorbic acid, β-carotene, total polyphenols contents and antioxidant activity are shown in Table 1.

Among the yellow varieties, the amount of ascorbic acid ranged between 0.80 mg g^−1^ (“Sassaniello”) and 1.72 mg g^−1^ (“Corno Marconi”) of fresh product. We observed a less wide situation in red varieties, where the concentration of ascorbic acid ranged between 0.82 mg g^−1^ (“Papaccella liscia) to 1.20 mg g^−1^ (“Corno Marconi”). Both in yellow and red varieties, “Corno Marconi” showed the highest amount of ascorbic acid. Among the red varieties is to remark also the high amount of ascorbic acid in the “Cornetto di Acerra” (1.19 mg g^−1^ of fresh product). The three green varieties showed the smallest content of ascorbic acid. “Friariello nocerese” contained a slightly higher content of ascorbic acid than “Friariello Napoletano” and “Friariello sigaretta”. For the most of the analyzed varieties, the amount of ascorbic acid was also higher than that reported by Howard et al. [19], and in some cases it was similar (in the case of yellow “Cazzone” or slightly inferior (red “Cazzone” and yellow “Corno di capra”) than reported by Mennella et al. [20]. Considering a portion of 100 g of raw pepper, all varieties of sweet pepper analyzed, with the exception of the two green varieties “Friariello Napoletano” and “Friariello a sigaretta”, reached a concentration of vitamin C at least two-fold the recommended daily dosage suggested by different international committees (WHO, the USA National Academy of Sciences, the USA Ministry of Health), and the *Codex alimentarius*, which indicate a minimum of 30 mg/die of ascorbic acid in human diet [21].

### 3.2. β-Carotene

β-Carotene or pro-vitamin-A is an abundant carotenoid present, as stable all-trans isomer, in many vegetal tissues including those of pepper. Due to its nutritional and coloring properties, it is used for several purposes, such as food coloring and preserving agent, in pharmaceuticals, as drug or drug ingredient that help to counteract vitamin A deficiency (VAD), thus preventing anomalies in growth, development, vision and immune function and cosmetics, for example as protective skin agent [22]. Pepper represents one of the primary dietary sources of pro-vitamin A [23] and generally, β-carotene is more abundant in fresh hot pepper than in sweet cultivars [23,24]. β-Carotene concentration is directly correlated to the total carotenoid content, being the precursor for the predominant orange and red carotenoids of pepper [25]. The data about the content of β-carotene present in the varieties of sweet pepper analyzed are shown in Table 1. In the yellow varieties, it ranged between 2.55 μg g^−1^ “(Corno Marconi”) and 5.79 μg g^−1^ of fresh product (“Cazzone giallo”); on the other hand, the red varieties exhibited always a content of β-carotene higher than the analogous yellow varieties, with values ranging from 3.96 μg g^−1^ (Sassaniello that therefore was the only variety to show a content of β-carotene inferior to the corresponding yellow “Sassaniello”) to 7.05 μg g^−1^ of fresh product (in the variety “Cazzone rosso”). Such values could be considered superior, for instance, respect to those reported by Thuphairo et al. [11], who indicated high values of carotenoids but considering the dry weight of the product. Respect to the shape of the varieties of peppers analyzed, we could say that the round red varieties of sweet pepper (“Papaccella napoletana” and “Papaccella napoletana liscia”) almost always had a higher β-carotene content than the analogous yellow varieties and that among the elongated varieties, “Cazzone” had a slightly greater β-carotene content respect to the others. The green varieties “Friariello a sigaretta” and “Friariello Napoletano” showed a lower content of β-carotene. Also in this case, “Friariello nocerese” exhibited a different behavior, with 2.24 μg of β-carotene g^−1^.

### 3.3. Total Polyphenol Content and Antioxidant Activity

Total polyphenol (TP) content ranged between 1.37 mmol g^−1^ (in the variety “Friariello sigaretta”) and 3.42 mmol g^−1^ (observed in the yellow “Papaccella napoletana”) of fresh product (Table 1). Such values were lower respect to those reported by Moktar et al. [26] and Hallmann and Rembiałkowska [27], but in some cases similar to those observed by Nazzaro et al. in elongated yellow and red varieties of sweet pepper cultivated in the Sicilia region [13] or to those cultivated in Romania, in different conditions of growth [28]. On the other hand, they resulted in some cases higher than those reported by Shotorbani et al. [29]. This indicates that such an important biochemical parameter might be related not only to the variety but also to the different geo-climatic conditions and to methods of treatment applied for their extraction. Among the round varieties of “Papaccella Napoletana”, the yellow type showed slightly higher content of TPs than the red type (3.42 and 3.13 mmol g^−1^ of fresh product, respectively); on the other hand, the difference of total polyphenols was more marked between the two “Papaccella liscia”, whose yellow type exhibited a TPs content 21.08% higher than the correspondent red type (2.80 mmol g^−1^ and 2.21 mmol g^−1^ of fresh product, respectively). Among the elongated varieties, the trend was different. Thus, while the red type of “Cazzone” contained less total polyphenols than the corresponding yellow type (2.66 mmol g^−1^ and 3.14 mmol g^−1^, respectively), the red “Corno Marconi” showed higher total polyphenols than the yellow type (3.36 mmol g^−1^ and 2.79 mmol g^−1^ of fresh product, respectively). A much more marked difference was observed within the variety “Corno di capra”, whose red type contained 1 mmol g^−1^ of total polyphenols more than the corresponding yellow type. Instead, the two varieties “Cornetto di Acerra” and “Sassaniello” showed almost the same amount of total polyphenols in both red and yellow types, with a very slight predominance in the yellow ones. The unusual difference between red and yellow “Corno di capra” was also observed in a genetic survey of the same set of traditional Campania peppers analyzed in the present study. The survey showed that the two varieties belong to two different and unrelated genetic groups according to Simple Sequence Repeats (SSR) DNA molecular markers (Tranchida-Lombardo et al., in prep.). Indeed, the “Corno di capra” yellow type is more related to the three green peppers, while the “Corno di capra” red type is more associated to “Cornetto di Acerra” (Tranchida-Lombardo et al., in prep.). The three green varieties of sweet pepper showed a much lower content of total polyphenols than those red and yellow. “Friariello nocerese” represented an exception, with total polyphenol similar to the yellow “Corno di capra” However, this did not correspond to a similar antioxidant activity, which resulted less effective (EC_50_: 7.27 versus 2.70, respectively) in “Friariello nocerese” than the “Corno di capra” that therefore exhibited higher amounts of β-carotene and ascorbic acid. Its content of TPs was also higher than that of the red “Papaccella liscia”. However, also in this case, to a higher amount of total polyphenols did not correspond a similar antioxidant activity; on the contrary, the antioxidant activity of red “Papaccella liscia” was two-time stronger respect to that of “Friariello nocerese” (EC_50_: 7.27 versus 3.64, respectively). The other two green peppers, “Friariello a sigaretta” and “Friariello Napoletano”, which showed the lowest values of all three parameters (ascorbic acid, β-carotene and total polyphenols) had the lowest effective antioxidant activity (EC_50_ = 9.57 mg and EC_50_ = 13.18 mg, respectively). Interestingly, the biochemical relationships observed among green peppers were also confirmed by SSR markers analysis, which showed that “Friariello nocerese” is less related to the two other green peppers (“Friariello sigaretta” and “Friariello napoletano”) and more related to the “Corno Marconi” variety (red and yellow types) (Tranchida-Lombardo et al. in prep.).

**Figure 2:**
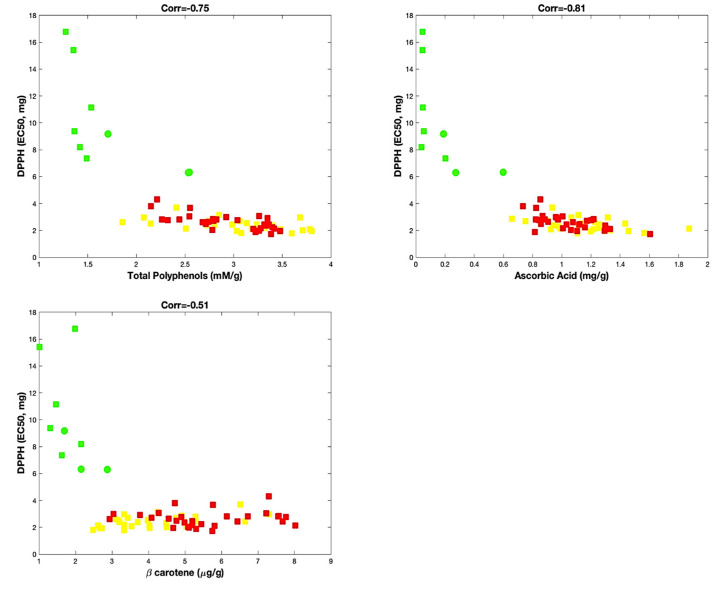
Correlation analysis. Red and yellow varieties are indicated with red and yellow squares, respectively. Green varieties are represented with green squares, except samples of “Friariello Nocerese”, represented by green circles, to highlight that at least two (of three samples) resulted very similar to red and yellow samples.

Statistical analysis, performed taking into account these biochemical parameters, allowed us to potentially identify the contribution of ascorbic acid, β-carotene and total polyphenols on the antioxidant activity. Thus, as shown in Figure 2, although all three parameters concurred to affect the antioxidant activity of the varieties of sweet pepper, the content of total polyphenols and ascorbic acid seemed better correlated with the antioxidant activity (corr = −0.75 and corr = −0.81, respectively) than the β-carotene (corr = −0.51).

In any case, for all analyses of correlation, we always observed a group formed by the red and yellow varieties and another distinct group formed by the green varieties. For this last group, the exception was represented by the “Friariello Nocerese”, which seemed most moved towards the red and yellow varieties. Therefore, the sweet pepper ‘Friariello’, widely cultivated in the Campania region, is also one of the most common Italian varieties [30], for which distinct group types were ascertained by Parisi et al. [31], on the basis of the genetic, morphological traits and some qualitative traits.

**Figure 3:**
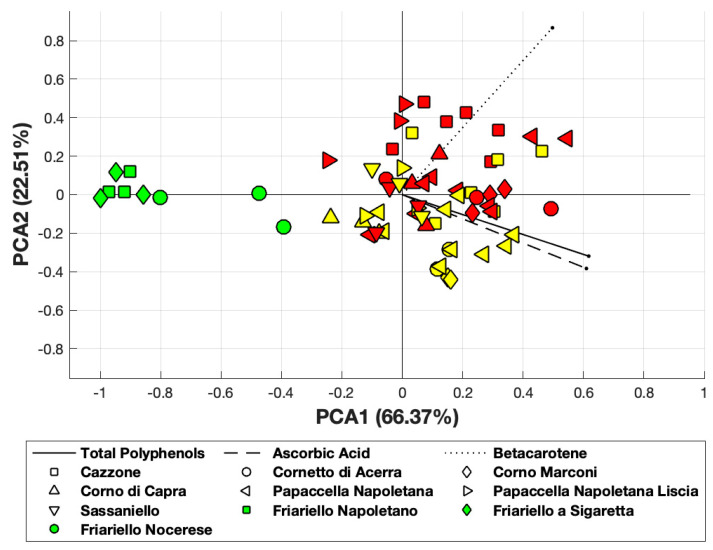
PCA obtained considering β-carotene, ascorbic and total polyphenols. Yellow and red-colored symbols indicate the yellow and red varieties, respectively.

Principal Component Analysis, obtained using MATLAB and applied to ascorbic acid, total polyphenols and β-carotene, revealed that the first principal component accounted for 66% of the total variance, while the first two components accounted for about 89% of the total variance of data (Figure 3). Interestingly, two of the green varieties (“Friariello napoletano” and “Friariello Sigaretta”, indicated as green squares) are well clustered and completely located near the PCA1 axis. On the other hand, we observed a significant overlapping between red and yellow varieties. The green variety “Friariello Nocerese” -indicated in the Figure 3 with green circled- showed, once again, greater similarity with the complex group of yellow and red varieties, in particular with the yellow “Corno di capra” than with the other two green varieties of sweet pepper.

### 3.4. Polyphenol Profile

Polyphenol profile, from qualitative and quantitative point of view, may vary in relation to genetic variation but also to different growth and geographic conditions. Obtaining as much information as possible on the polyphenolic composition becomes crucial for identifying the best varieties, from a qualitative and health-nutritional point of view, existing in a given territory. As far as we know, there are no studies that have characterized such a large number of traditional Campania varieties from a biochemical point of view. In particular, there are no scientific studies reporting the analysis of the qualitative and quantitative profile of the polyphenols present in many of the traditional sweet pepper varieties of the Campania region. This study was carried out using UPLC, an analytical approach that proved to be a powerful method for the analysis of individual polyphenols in a few minutes (in our case in an analysis time not exceeding 15’) and capable of providing a detailed analysis of the phenolic molecules and, above all, of their concentration, contained in the varieties. All data, reported as μg g^−1^ respect to the metabolites identified through UPLC are shown in Table 2.

Gallic acid and chlorogenic acid were the most abundant phenolic acids in all varieties. Among the yellow and red varieties, gallic acid ranged between 44.80 μg g^−1^ (in the red “Papaccella liscia”) and 185.70 μg g^−1^ (found in the yellow type of “Corno Marconi”). “Friariello Nocerese” showed a content of gallic acid more similar, in terms of concentration, to some of yellow and red varieties (such as to the red variety of “Papaccella napoletana” (117.34 μg g^−1^ and 112.27 μg g^−1^, respectively), than to the other two green varieties of Friariello, which content did not exceed 69.26 μg g^−1^ (“Friariello napoletano”). In general, almost all the traditional varieties analyzed exhibited a content of gallic acid undoubtedly superior if compared to that present in some commercial varieties such as Roberta or Berceo [27,32], but similar to such varieties for the high content of chlorogenic acid, the low amount of caffeic acid and for the negligible quantity of apigenin, therefore present only in very few varieties. Chlorogenic acid showed a sharped distribution along the varieties. It is to highlight that the green variety “Friariello Nocerese” contained the highest amount of this metabolite (273 μg g^−1^); on the contrary, we observed that the yellow type of “Cornetto di Acerra” showed a ninth of chlorogenic acid respect to “Friariello Nocerese”. Probably in the “Cornetto di Acerra” other metabolites, not recognized with the available standards, were also present, justifying the amount of polyphenols that, in the yellow “Cornetto di Acerra”, was 1mmol higher than “Friariello Nocerese”. Caffeic acid was much less abundant both in yellow and red varieties, not exceeding 77.11 μg g^−1^ (in the yellow “Corno di Capra”), while it was enough abundant in the three varieties of green pepper, reaching also 97.10 μg g^−1^ in the “Friariello napoletano”. *p*-Coumaric acid was absent in all varieties, except two green varieties (“Friariello Napoletano and “Friariello Nocerese”), which content of this metabolite did not exceeded 8.06 μg g^−1^. The trend of caffeic acid and chlorogenic acid was in agreement with Blanco-Rios et al. [33], which, analyzing different varieties of Mexican sweet pepper, found a higher amount of caffeic acid and chlorogenic acid in the green varieties, with a decreasing quantity starting from the red varieties, and following towards the orange and yellow ones. The almost complete absence of p-coumaric acid, observed only in the green varieties of “Friariello” is in agreement with Dimitriu et al. [34], which led to an increase of this metabolite at to 0.0487 mg GAE in 100 g FW only under microorganism fertilization in cultivars of pepper where it was not detected. Among flavonoids, catechin was detected in all varieties (except in the yellow “Corno Marconi” and in the red “Papaccella liscia”) reaching also 166.68 μg g^−1^ (in the red “Sassaniello”). Its isomer epicatechin was slightly less abundant than catechin (121.93 μg g^−1^ as the highest concentration, in the red “Sassaniello”). The content of catechin observed by us was certainly higher than that reported by Ghasemnezhad et al. [35] in some varieties of sweet pepper, which contained a concentration of this metabolite ranging between 3.2 μg g^−1^ FW and 15.54 μg g^−1^ FW. The presence of such an important amount of catechin and epicatechin in some varieties of sweet pepper is very important. Catechins can reduce the risk of occurrence and development of some diseases. These metabolites can concur to regulate the glucose/insulin and lipid metabolism [36]; they have also neuroprotective [37], hearth protective [38], and anti-inflammatory effects [39]. Catechin can also protect from eye macula [40]. Catechin has also antibacterial activity. Nazzaro et al. found a strict correlation between the presence of catechin in extra virgin olive oil (EVO) and antibacterial activity exhibited by the EVO extract against *Staphylococcus aureus* [41]. Recently, catechin extracted from green tea-based polyphenol was also associated to rare earth (RE) metal ions to prepare catechin–RE complexes that showed significant anti-biofilm properties against *Pseudomonas aeruginosa*, *Staphylococcus sciuri* and *Aspergillus niger*, acting through the damage of microbial cell membrane [42]. Epicatechin and its metabolites can enhance muscle performance, improve symptoms of cardiovascular and cerebrovascular diseases, and support human health preventing diabetes and protecting the nervous system [43]. The presence of a so high amount of catechins and epicatechin in the varieties of sweet pepper resulted still more interesting, taking into account that, in some cases, flavonoid contents of pepper extracts can be enhanced with thermal process, such as by increasing temperature to 65 °C [29].

Quercetin was detected in the varieties “Papaccella napoletana” (both yellow and red), “Corno Marconi” (both yellow and red, 17.23 μg g^−1^ and 124.86 μg g^−1^, respectively), in the red “Cornetto di Acerra” (104.35 μg g^−1^) and in the red “Corno di Capra” (that showed 7.36 μg g^−1^). Also in this case, our results, at least for the green varieties, were in agreement with Blanco-Rois et al. [33]; however, unlike these last, we found a certain amount of quercetin also in some varieties of red and yellow sweet peppers. Quercetin is one of the metabolites with the most known healthy effects. Recently Pingili et al. [44] ascertained an important role of quercetin as liver protective agent against different drugs and toxic agents. Therefore, due to its healthy properties [45], quercetin has been also used in the formulation of novel functional foods, such as quercetin-fortified bread [46]. Yellow “Papaccella napoletana” exhibited a content of quercetin also superior respect to that observed by Ghasemnezhad et al. in the variety Arona [35]. Rutin was detected in some varieties, such as “Cazzone” (both yellow and red), “Papaccella napoletana” (both yellow and red), and in the red types of “Cornetto di Acerra” and “Sassaniello”. It was also identified and quantified in the two green varieties of sweet pepper “Friariello napoletano” and “Friariello sigaretta” that, on the other hand did not contain naringenin, present only in the third variety of green pepper “Friariello nocerese” and detected also in other two varieties, the yellow “Corno di capra” and the red “Sassaniello”.

From a global analysis of polyphenolic profile, we could observe that the red “Sassaniello” and the yellow “Papaccella napoletana” showed almost all metabolites, except *p*-coumaric acid and quercetin in the “Sassaniello” and *p*-coumaric acid and naringenin in the “Papaccella napoletana”. Therefore, the red type “Papaccella liscia” missed even seven polyphenols, in particular caffeic acid among the phenolic acids, and almost all flavonoids except epicatechin. This clearly shows in general a different metabolic pathway between the varieties of sweet pepper, which affects the presence and amount of these secondary metabolites.

**Figure 4:**
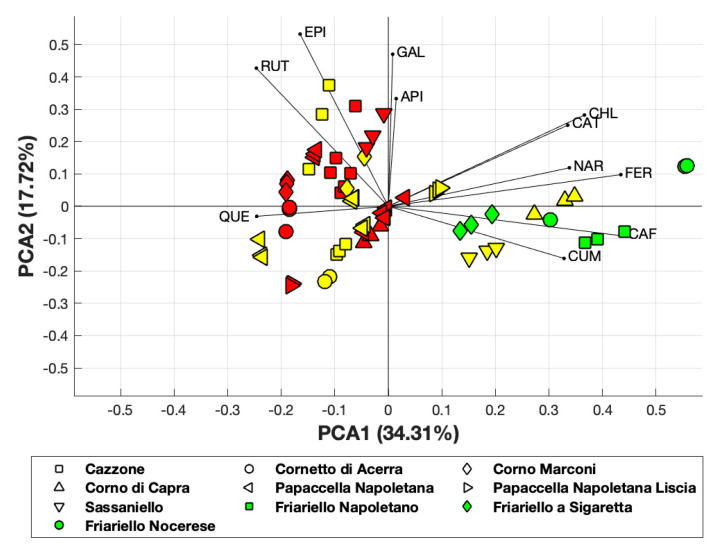
PCA obtained considering polyphenol composition. Yellow and red-colored symbols indicate the yellow and red varieties, respectively.

PCA applied to the polyphenol composition of all varieties of sweet pepper analyzed, revealed that the first principal component accounted for 34.31% of the total variance, while the first two components accounted for about 52% of the total variance (Figure 4).

Although PCA covered 52% of the total variance, it allowed us to observe the clustering of several yellow and red varieties and that the yellow varieties “Corno di capra” and “Sassaniello” were close to the green varieties of “Friariello”.

**Figure 5:**
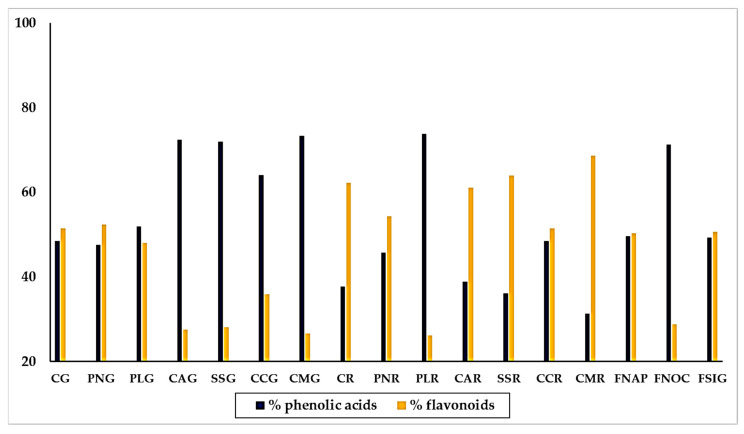
Distribution of phenolic acids and flavonoids and phenolic acids, respect to the molecules recognized by UPLC, in yellow, red and green traditional varieties of sweet pepper of the Campania Region. Varieties = CG: Cazzone, yellow; PNG: Papaccella napoletana, yellow; PLG: Papaccella liscia, yellow; CAG: Cornetto di Acerra, yellow; SG: Sassaniello, yellow; CCG: Corno di Capra, yellow; CMG: Corno Marconi, yellow; CR: Cazzone, red; PNR: Papaccella napoletana, red; PLR: Papaccella liscia, red; CAR: Cornetto di Acerra, red; SR: Sassaniello, red; CCR: Corno di Capra, red; CMR: Corno Marconi, red; FNAP: Friariello Napoletano, green; FNOC: Friariello Nocerese, green; FSIG: Friariello Sigaretta, green.

Calculating the percentages of the individual polyphenols with respect to the sum of the polyphenols recognized by the chromatographic analysis, and assembling phenolic acids and flavonoids in two groups, we could observe the general distribution of acidic phenols and flavonoids among the varieties of sweet pepper. Results are shown in Figure 5.

By the whole, the composition exhibited by the yellow and red varieties was quite different. Thus, we found that the majority of yellow varieties contained much more phenolic acids than flavonoids, in particular “Corno Marconi” (73.34% phenolic acids and 26.66% flavonoids), “Cornetto di Acerra” (72.5% phenolic acids and 27.5% flavonoids), “Sassaniello” (71.9% phenolic acids and 28.1% flavonoids) and “Corno di capra” (64.1% phenolic acids and 35.9% flavonoids). In the varieties “Cazzone” and” Papaccella liscia” the percentage of phenolic acids and flavonoids was almost equal, with a slightly preponderance of flavonoids. The trend was completely opposite in the red varieties, which contained generally much more flavonoids than phenolic acids. This was the behavior of “Corno Marconi (68.9% flavonoids and 31.31% phenolic acids), “Sassaniello” (containing 63.91% flavonoids and 36.09% phenolic acids) and “Cazzone” (62.68% flavonoids and 37.72% phenolic acids). Two red varieties, “Papaccella napoletana” and “Corno di capra” showed a similar content of phenolic acids and flavonoids. Only “Papaccella liscia” contained much more phenolic acids (73.81%) than flavonoids (26.19%). The three varieties of “Friariello” behaved completely different from each other. Thus, while the “Friariello Nocerese” showed a trend more similar to that of the yellow varieties, in particular “Cornetto di Acerra” and “Sassaniello”, with a content of acid phenols (71.22%) much higher than that of the flavonoids (28.78%). The “Friariello napoletano” and the “Friariello a sigaretta” instead practically the same percentage of acid phenols (49.63% and 49.33%, respectively) and flavonoids (50.36% and 50.67% respectively). The behavior of “Friariello Nocerese”, which in general showed highest values of phenolic acids and flavonoids than the other two green varieties, was in agreement with the results of Mennella et al. [20], which found similarities between ‘Friariello Nocerese’ and some yellow varieties of sweet pepper, in spite of the clear differences that the two varieties show as regard their morphological traits and color at maturation.

## 4. Conclusions

Breeding for high-value vegetables is an increasingly preferred ambition and the enhancement of crop bioactive compounds might be strategic to encounter the actual ideas of the market and consumers indeed. As far as we know, this is the first time that a similar study was performed on different yellow, red and green traditional varieties of sweet pepper of the Campania region, grown at the same conditions and collected in the same period, so to avoid as more as possible all those variables that could affect the metabolic pathway of these products [47]. Our results suggest the great potential of these landraces in terms of phytochemicals of health interest. The interrelationships between the parameters analyzed and the different varieties let us to light on a clustering of several red and yellow varieties, and that mainly the yellow ”Corno di capra” was closer to the green varieties of “Friariello”. This could contribute in the exploitation and improvement of both cultivation and breeding programs at regional, national and international level, with the aims to safeguard the crop biodiversity, taking into account the health of consumers and without forgetting that an increase in the cultivation of these traditional varieties could rise the farmers’ income.

## Figures and Tables

**Table 1:** Content of ascorbic acid, β-carotene, total polyphenols and antioxidant activity exhibited by the yellow, red and green traditional varieties of sweet pepper of the Campania region. Data represent the average (± SD) of three independent experiments. For details, see the Section Materials and Methods.

Table 1	Ascorbic Acid mg g^−1^ ± SD	β-Carotene μg g^−1^ SD	Total Polyphenols mMol g^−1^ ± SD	Antioxidant Activity mg EC_50_ ± SD
Yellow varieties				
Cazzone giallo	1.13 ± 0.13	5.79 ± 1.24	3.14 ± 0.43	2.74 ± 0.51
Papaccella Napoletana gialla	1.12 ± 0.15	3.84 ± 0.73	3.42 ± 0.40	2.10 ± 0.21
Papaccella liscia gialla	0.95 ± 0.15	4.33 ± 0.93	2.80 ± 0.05	2.88 ± 0.24
Corno Marconi giallo	1.72 ± 0.22	2.55 ± 0.09	2.79 ± 0.40	1.97 ± 0.22
Corno di capra giallo	1.33 ± 0.10	3.20 ± 0.12	2.03 ± 0.15	2.70 ± 0.24
Cornetto di Acerra giallo	1.35 ± 0.14	2.99 ± 0.47	3.18 ± 0.21	2.06 ± 0.16
Sassaniello giallo	0.80 ± 0.17	4.56 ± 0.47	3.02 ± 0.19	2.56 ± 0.38
Red varieties				
Cazzone rosso	1.02 ± 0.17	7.05 ± 0.79	2.66 ± 0.35	2.93 ± 0.42
Papaccella Napoletana rossa	1.13 ± 0.16	5.38 ± 1.59	3.13 ± 0.29	2.26 ± 0.28
Papaccella liscia rossa	0.82 ± 0.08	6.25 ± 1.35	2.21 ± 0.06	3.64 ± 0.76
Corno Marconi rosso	1.20 ± 0.12	5.31 ± 0.58	3.36 ± 0.14	2.11 ± 0.14
Corno di capra rosso	0.93 ± 0.07	4.94 ± 1.19	3.07 ± 0.26	2.84 ± 0.08
Cornetto di Acerra rosso	1.19 ± 0.38	5.24 ± 0.49	3.15 ± 0.38	2.22 ± 0.43
Sassaniello rosso	0.91 ± 0.05	3.96 ± 0.80	2.98 ± 0.26	2.91 ± 0.23
Green varieties				
Friariello Napoletano	0.05 ± 0.01	1.64 ± 0.45	1.44 ± 0.09	9.57 ± 1.50
Friariello Nocerese	0.35 ± 0.22	2.24 ± 0.59	2.26 ± 0.48	7.27 ± 1.65
Friariello sigaretta	0.10 ± 0.09	1.54 ± 0.49	1.37 ± 0.11	13.18 ± 5.09

**Table 2:** Polyphenol composition of the yellow, red and green traditional varieties of sweet pepper of the Campania region, obtained by UPLC analysis, expressed as μg g^−1^ ± Standard Deviation.

	GAL	CHL	CAF	CUM	FER	CAT	EPI	QUE	RUT	API	NAR
CG	127.81 ± 20.85	98.66 ± 26.13	19.46 ± 5.12	0 ± 0.00	0 ± 0.00	98.93 ± 7.20	112.09 ± 12.65	0 ± 0.00	49.91 ± 5.22	0 ± 0.00	0 ± 0.00
PNG	100.16 ± 21.20	97.79 ± 27.77	23.13 ± 5.48	0 ± 0.00	7.01 ± 1.63	95.51 ± 4.59	27.91 ± 2.71	76.38 ± 2.51	46.56 ± 3.65	4.71 ± 0.78	0 ± 0.00
PLG	109.83 ± 2.10	114.88 ± 2.20	51.47 ± 0.98	0 ± 0.00	22.78 ± 0.44	130.68 ± 2.50	145.96 ± 2.79	0 ± 0.00	0 ± 0.00	0 ± 0.00	0 ± 0.00
CAG	70.96 ± 4.64	31.00 ± 2.03	12.51 ± 0.82	0 ± 0.00	7.54 ± 0.49	44.10 ± 2.88	2.33 ± 0.33	0 ± 0.00	0 ± 0.0)	0 ± 0.00	0 ± 0.00
SG	100.13 ± 6.44	115.29 ± 7.41	29.16 ± 1.87	6.29 ± 0.40	23.44 ± 1.51	107.05 ± 6.88	0 ± 0.00	0 ± 0.00	0 ± 0.00	0 ± 0.00	0 ± 0.00
CCG	97.17 ± 7.19	127.05 ± 9.40	77.11 ± 5.71	0 ± 0.00	24.58( ± 1.82	145.94 ± 10.80	0 ± 0.00	0 ± 0.00	0 ± 0.00	13.58 ± 1.01	22.97 ± 1.70
CMG	185.70 ± 27.89	103.93 ± 15.05	15.48 ± 2.24	0 ± 0.00	18.73 ± 2.71	0 ± 0.00	92.56 ± 13.40	17.23 ± 2.50	0 ± 0.00	7.82 ± 1.13	0 ± 0.00
CR	96.94 ± 20.85	97.79 ± 27.77	3.09 ± 0.73	0 ± 0.00	14.09 ± 1.68	129.18 ± 13.08	110.84 ± 15.15	0 ± 0.00	101.55 ± 18.69	8.32 ± 1.33	0 ± 0.00
PNR	112.27 ± 17.05	119.74 ± 20.11	15.69 ± 1.68	0 ± 0.00	10.37 ± 1.22	130.37 ± 11.53	92.47 ± 4.97	50.70 ± 2.23	33.02 ± 4.95	0 ± 0.00	0 ± 0.00
PLR	44.80 ± 1.13	58.54 ± 1.48	0 ± 0.00	0 ± 0.00	0.44 ± 0.01	0 ± 0.00	36.81 ± 0.93	0 ± 0.00	0 ± 0.00	0 ± 0.00	0 ± 0.00
CAR	93.74 ± 11.29	81.01 ± 9.70	3.82 ± 0.46	0 ± 0.00	11.10 ± 1.33	52.46 ± 6.28	64.03 ± 7.67	104.35 ± 12.50	77.24 ± 9.25	0 ± 0.00	0 ± 0.00
SR	101.18 ± 9.01	103.51 ± 9.22	4.49 ± 0.40	0 ± 0.00	10.02 ± 0.89	166.68 ± 14.84	121.93 ± 10.86	0 ± 0.00	64.41 ± 5.74	29.61 ± 2.64	5.44 ± 0.48
CCR	85.87 ± 7.39	65.53 ± 5.64	29.31 ± 2.52	0 ± 0.00	6.16 ± 0.53	133.93 ± 11.52	52.68 ± 4.53	7.36 ± 0.63	0 ± 0.00)	4.05 ± 0.35	0 ± 0.00
CMR	90.80 ± 3.83	108.05 ± 4.56	0 ± 0.00	0 ± 0.00	7.35 ± 0.31	119.05 ± 5.03	99.16 ± 4.19	124.86 ± 5.27	109.27 ± 4.61	0 ± 0.00	0 ± 0.00
FNAP	69.26 ± 4.19	112.93 ± 6.84	97.10 ± 5.88	8.06 ± 0.49	29.62 ± 1.79	244.37 ± 14.80	67.86 ± 4.11	0 ± 0.00	9.46 ± 0.57	0 ± 0.00	0 ± 0.00
FNOC	117.34 ± 24.94	273.57 ± 28.15	55.66 ± 1.83	2.71 ± 0.58	30.85 ± 2.56	170.79 ± 16.30	0 ± 0.00	0 ± 0.00	0 ± 0.00	0 ± 0.00	23.19 ± 1.93
FSIG	65.95 ± 5.20	124.66 ± 9.88	44.88 ± 3.56	0 ± 0.00	25.97 ± 2.06	216.03 ± 17.13	0 ± 0.00	0 ± 0.00	52.47 ± 4.17	0 ± 0.00	0 ± 0.00

Legend: molecules = GAL: gallic acid; CHL: chlorogenic acid; CAF: caffeic acid; CUM: *p*-coumaric acid; FER: ferulic acid; CAT: catechin; EPI: epicatechin; QUE: quercetin; RUT: rutin; NAR: naringenin; API: apigenin; Varieties = CG: Cazzone, yellow; PNG: Papaccella napoletana, yellow; PLG: Papaccella liscia, yellow; CAG: Cornetto di Acerra, yellow; SG: Sassaniello, yellow; CCG: Corno di Capra, yellow; CMG: Corno Marconi, yellow; CR: Cazzone, red; PNR: Papaccella napoletana, red; PLR: Papaccella liscia, red; CAR: Cornetto di Acerra, red; SR: Sassaniello, red; CCR: Corno di Capra, red; CMR: Corno Marconi, red; FNAP: Friariello Napoletano, green; FNOC: Friariello Nocerese, green; FSIG: Friariello Sigaretta, green.
